# Supplementary material for: A Global Screen for Assembly State Changes of the Mitotic Proteome by SEC-SWATH-MS
Source: Cell Syst. 2020 Feb 26;10(2):133–155.e6. doi: 10.1016/j.cels.2020.01.001 (PMC7042714; doi:10.1016/j.cels.2020.01.001)

Q9Y676 | RT18B_HUMAN | MRPS18B C6orf14 HSPC183 PTD017
Monomer MW [kDa]: 29.396 Monomer expected elution fraction: 51

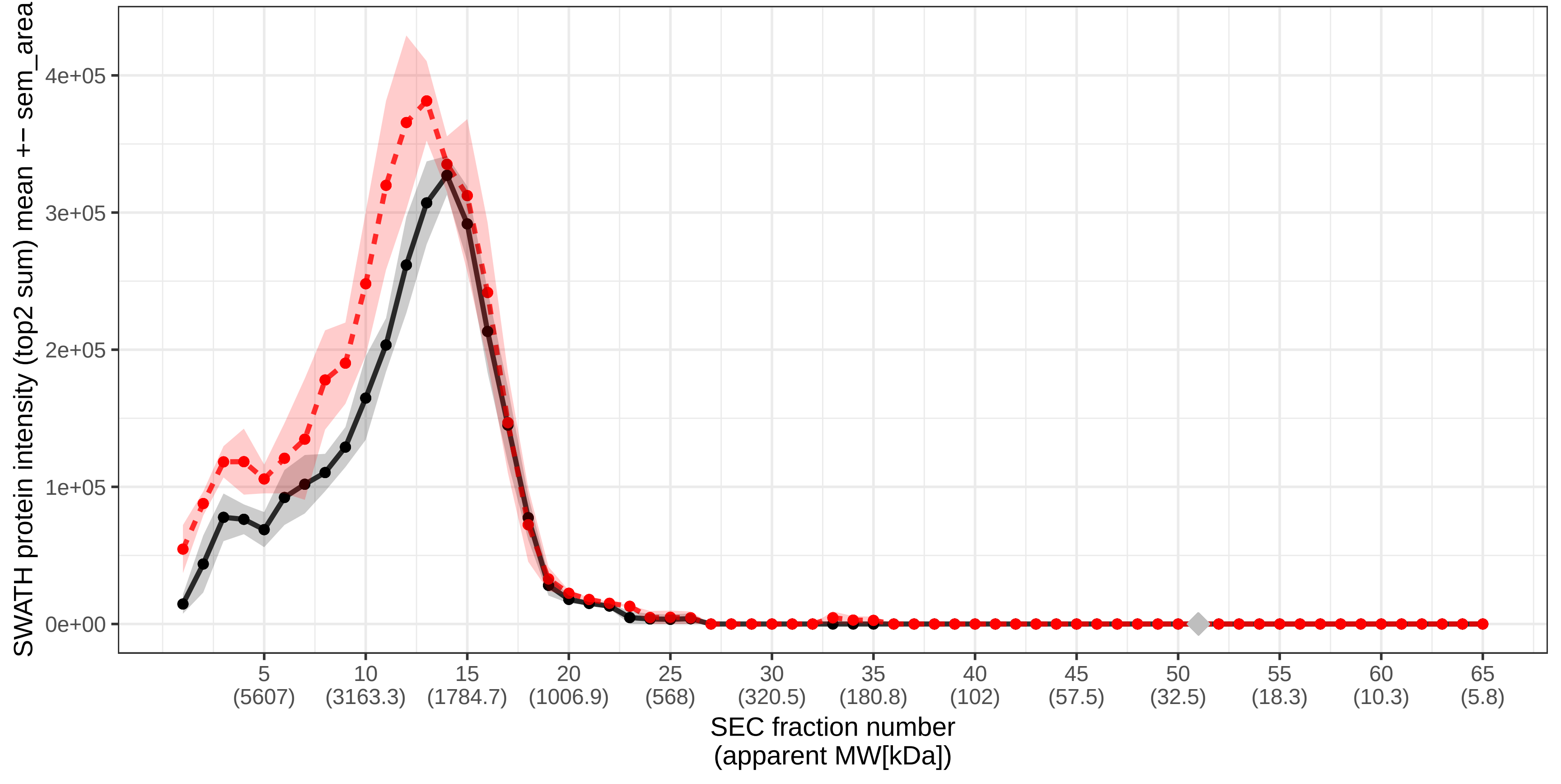

Supplement: Data S1. SEC-SWATH-MS Protein Chromatograms, Related to Figure 1 [file mmc6.zip › SECchrom_Q9Y676_RT18B_HUMAN_MRPS18B_C6orf14_HSPC183_PTD017.pdf]
